# Supplementary material for: Fluent molecular mixing of Tau isoforms in Alzheimer’s disease neurofibrillary tangles
Source: Nat Commun. 2022 May 27;13:2967. doi: 10.1038/s41467-022-30585-0 (PMC9142584; doi:10.1038/s41467-022-30585-0)
Supplement: Supplementary file 1 — Supplementary Information [file 41467_2022_30585_MOESM1_ESM.pdf]

Supplementary Information

Fluent Molecular Mixing of Tau Isoforms in Alzheimer's Disease Neurofibrillary Tangles

Aurelio J. Dregni [1$], Pu Duan[1$], Hong Xu[2], Lakshmi Changolkar[2], Nadia El Mammeri[1], Virginia M.-Y. Lee[2], and Mei Hong[1]*

[1] Department of Chemistry, Massachusetts Institute of Technology, 170 Albany Street, Cambridge, MA 02139

[2] Department of Pathology and Laboratory Medicine, Institute On Aging and Center for Neurodegenerative Disease Research, University of Pennsylvania School of Medicine, Philadelphia, PA, 19104, USA

This PDF file includes:
Supplementary Tables 1 - 5
Supplementary Figures 1 to 7

Supplementary Table 1. Cases of Alzheimer's disease brains used in this solid-state NMR study.

**Case no.**	**Disease Duration (yr)**	**Gender**	**Neuropathological diagnosis**
1	11	F	Alzheimer's disease
2	6	F	Alzheimer's disease
3	7	M	Alzheimer's disease
4	9	F	Alzheimer's disease
5	11	F	Alzheimer's disease
6	9	M	Alzheimer's disease
7	8	M	Alzheimer's disease
8	16	F	Alzheimer's disease
9	3	M	Alzheimer's disease
10	8	F	Alzheimer's disease

Supplementary Table 2. Characterization of supernatant fraction from human brain extraction (“AD Seeds”).

	Conc. of Tau by ELISA	Total protein Conc. by BCA	Purity (tau / total protein)	Conc. of Aβ_{42} by ELISA	Conc. of Aβ_{42} by ELISA	Conc. of α-synuclein by ELISA
Pooled AD-tau	0.55 μg/μl	5.28 μg/μl	10%	14.61 ng/μl	59.16 ng/μl	0.46 ng/μl

Supplementary Table 3. Morphologies of AD-seeded tau and heparin-fibrilized tau. At least 5 different particles from two or more different TEM images were sampled using ImageJ, and a total of at least 10 readings were averaged to give each of the statistics below.

Samples	Twisted fibrils			Straight fibrils
	Crossover length	Narrow width	Wide width	Width
[AD]4R^{N}3R^C	86.9 ± 10.6 nm	9.9 ± 1.6 nm	25.1 ± 1.8 nm	15.2 ± 0.9 nm
[AD]4R^{NC}	99.3 ± 9.4 nm	10.7 ± 1.6 nm	24.3 ± 1.7 nm	15.9 ± 1.3 nm
[AD]3R^{NC}	100.7 ± 16.5 nm	10.6 ± 1.0 nm	24.7 ± 2.7 nm	16.3 ± 1.0 nm
[AD]3R^{N}4R^C	93.8 ± 18.0 nm	10.0 ± 1.1 nm	25.5 ± 1.9 nm	16.0 ± 1.0 nm
Heparin-0N4R	16.1 ± 1.6 nm, long straight filaments			
Heparin-0N3R	22.9 ± 0.6 nm, straight ribbons			
Hep-0N3R/0N4R mix	13.8 ± 1.3 nm, long straight filaments			

Supplementary Table 4. Concentrations of tau added to fibril growth mixtures and validation by the 1D NMR spectral quantification.

**Samples**	**Tau monomers for fibrillization**	**Composition of AD seeded samples from 1D NMR spectral quantification**
[AD]4R^{N}3R^C	18 μM ^{2}H,^{13}C-0N3R + 18 μM ^{2}H,^{15}N-0N4R	#1: 40.0% 3R, 60.0 ± 3.0% 4R,<br>#2: 37.4% 3R, 62.6 ± 3.0% 4R
[AD]4R^{NC}	36 μM ^{2}H,^{13}C,^{15}N-0N4R	100% 4R
[AD]3R^{NC}	36 μM ^{2}H,^{13}C,^{15}N-0N3R	100% 3R
[AD]3R^{N}4R^C	18 μM ^{2}H,^{13}C-0N4R + 18 μM ^{2}H,^{15}N-0N3R	#1: 39.2 ± 3.0% 3R, 60.8% 4R<br>#2: 37.9 ± 3.0% 3R, 62.1% 4R
100:0 4R	10 μM ^{2}H,^{13}C-0N4R + 0 μM ^{2}H,^{15}N-0N4R	
70:30 4R	7 μM ^{2}H,^{13}C-0N4R + 3 μM ^{2}H,^{15}N-0N4R	
60:40 4R	6 μM ^{2}H,^{13}C-0N4R + 4 μM ^{2}H,^{15}N-0N4R	
50:50 4R	5 μM ^{2}H,^{13}C-0N4R + 5 μM ^{2}H,^{15}N-0N4R	
40:60 4R	4 μM ^{2}H,^{13}C-0N4R + 6 μM ^{2}H,^{15}N-0N4R	
30:70 4R	3 μM ^{2}H,^{13}C-0N4R + 7 μM ^{2}H,^{15}N-0N4R	

Supplementary Table 5. NMR experimental parameters in this study. All experiments were conducted using a Bruker 1.3 mm HCN probe on a Bruker Avance III HD 600 MHz spectrometer or Avance II 800 MHz spectrometer. The rf field strength for ^{1}H, ^{13}C and ^{15}N channel pulses are 83.3 kHz, 62.5 kHz and 50 kHz, respectively.

Experiment	NMR Parameters	Experimental Time per sample
1D ^{13}C DP	B_0 = 14.1 T, ns = 2048 or 6144, τ_{rd} = 5 s, τ_{dw} = 10 μs, τ_{acq} = 30.7 ms, $\nu_{H,DEC}$ = 130 kHz, ν_{MAS} = 20 kHz, $T_{bearing}$ = 278 K, δ_{H2O} = 4.89 ppm, d1 = 5 s	3 hr or 9 hr
1D J-hnH	B_0 = 14.1 T, ns = 256 or 512, τ_{rd} = 1.5 s, τ_{dw} = 15 μs, τ_{acq} = 92.2 ms, τ_{INEPT} = 10 ms, $\tau_{MISSISSIPPI}$ = 0.3 s, $\nu_{H,DEC}$ = 130 kHz, $\nu_{C,DEC}$ = 3.6 kHz, $\nu_{N,DEC}$ = 3.6 kHz, ν_{MAS} = 20 kHz, $T_{bearing}$ = 293 K, δ_{H2O} = 4.77 ppm.	10 min or 20 min
1D dipolar hnH	B_0 = 14.1 T, ns = 256 or 512, τ_{rd} = 1.5 s, τ_{dw} = 15 μs, τ_{acq} = 23.0 ms, τ_{HN} = 1250 μs, τ_{NH} = 400 μs, $\tau_{MISSISSIPPI}$ = 0.3 s, $\nu_{C,DEC}$ = 3.6 kHz, $\nu_{N,DEC}$ = 3.6 kHz, ν_{MAS} = 20 kHz, $T_{bearing}$ = 278 K, δ_{H2O} = 4.89 ppm.	10 min or 20 min
^{1}H-detected ^{15}N-^{13}C REDOR	B_0 = 14.1 T, ns = 256*6*2 to 256*8*2 for each REDOR time, τ_{rd} = 1.5 s, τ_{dw} = 15 μs, τ_{acq} = 23.0 ms, τ_{HN} = 1250 μs, τ_{NH} = 400 μs, $\tau_{MISSISSIPPI}$ = 0.3 s, τ_{REDOR} = 0.1 ms to 25 ms (28 REDOR time in total), $\nu_{H,DEC}$ = 130 kHz, $\nu_{C,DEC}$ = 3.6 kHz, $\nu_{N,DEC}$ = 3.6 kHz, ν_{MAS} = 20 kHz, ν_C = 62.5 kHz, $T_{bearing}$ = 278 K, δ_{H2O} = 4.89 ppm.	2-3 days per sample
2D J-hNH	B_0 = 14.1 T, ns = 64 to 160, τ_{rd} = 1.5 s, τ_{dw} = 15 μs, τ_{acq} = 92.2 ms, $t_{1,inc}$ = 274 μs, $t_{1,max}$ = 70.2 ms, τ_{INEPT} = 10 ms, $\tau_{MISSISSIPPI}$ = 0.3 s, $\nu_{H,DEC}$ = 130 kHz, $\nu_{C,DEC}$ = 3.6 kHz, $\nu_{N,DEC}$ = 3.6 kHz, ν_{MAS} = 20 kHz, $T_{bearing}$ = 293 K, δ_{H2O} = 4.77 ppm.	~2 days per sample
2D dipolar hNH	B_0 = 18.8 T (Heparin-4R^{NC}, [AD]4R^{NC}) or 14.1 T (others), ns = 400 to 600, τ_{rd} = 1.5 s, τ_{dw} = 15 μs, τ_{acq} = 38.4 ms, $t_{1,inc}$ = 225 μs (Heparin-4R^{NC}, [AD]4R^{NC}) or 300 μs (others), $t_{1,max}$ = 30 ms, τ_{HN} = 1250 μs, τ_{NH} = 400 μs, $\tau_{MISSISSIPPI}$ = 0.3 s, $\nu_{H,DEC}$ = 10 kHz, $\nu_{C,DEC}$ = 10 kHz, $\nu_{N,DEC}$ = 10 kHz, ν_{MAS} = 55 kHz, $T_{bearing}$ = 250 K, δ_{H2O} = 4.89 ppm.	2-3 days per sample
3D hCANH	B_0 = 14.1 T, ns = 24, τ_{rd} = 1.5 s, τ_{dw} = 15 μs, τ_{acq} = 38.4 ms, $t_{1,inc}$ = 160 μs, $t_{1,max}$ = 4.8 ms, $t_{2,inc}$ = 300 μs, $t_{2,max}$ = 10.5 ms, τ_{HC} = 2 ms, τ_{CN} = 8 ms, τ_{NH} = 400 μs, $\tau_{MISSISSIPPI}$ = 0.2 s, $\nu_{H,DEC}$ = 10 kHz, $\nu_{H,CN}$ = 0 kHz, $\nu_{C,DEC}$ = 10 kHz, $\nu_{N,DEC}$ = 10 kHz, ν_{MAS} = 55 kHz, $T_{bearing}$ = 247 K, δ_{H2O} = 4.89 ppm.	2 days
3D hCA(co)NH	B_0 = 14.1 T, ns = 40, τ_{rd} = 1.5 s, τ_{dw} = 15 μs, τ_{acq} = 23.0 ms, $t_{1,inc}$ = 160 μs, $t_{1,max}$ = 4.8 ms, $t_{2,inc}$ = 300 μs, $t_{2,max}$ = 10.5 ms, τ_{HC} = 2 ms, τ_{DREAM} = 8 ms, τ_{CN} = 8 ms, τ_{NH} = 400 μs, $\tau_{MISSISSIPPI}$ = 0.2 s, $\nu_{H,DEC}$ = 10 kHz, $\nu_{H,CN}$ = 0 kHz, $\nu_{C,DEC}$ = 10 kHz, $\nu_{N,DEC}$ = 10 kHz, ν_{MAS} = 55 kHz, $T_{bearing}$ = 247 K, δ_{H2O} = 4.89 ppm.	3.5 days

Definitions of symbols: B_0 = magnetic field; ns = number of scans per free induction decay; τ_{rd} = recycle delay; $t_{1,max}$ = maximum t_1 evolution time; $t_{1,inc}$ = t_1 increment; $t_{2,max}$ = maximum t_2 evolution time; $t_{2,inc}$ = t_2 increment; τ_{dw} = dwell-time in the direct dimension; τ_{acq} = maximum acquisition time in the direct dimension; $\nu_{X,DEC}$ = X channel (^{1}H, ^{13}C or ^{15}N) rf field strength for decoupling during acquisition; $\nu_{H,CN}$ = ^{1}H rf field strength for decoupling during ^{13}C-^{15}N SPECIFIC-CP; $T_{bearing}$ = thermocouple-reported bearing gas temperature; δ_{H2O} = ^{1}H chemical shift of water; ν_{MAS} = MAS frequency; τ_{HX} = ^{1}H-X cross polarization contact time; τ_{CN} = ^{13}C-^{15}N SPECIFIC-CP contact time; τ_{DREAM} = ^{13}C-^{13}C DREAM contact time; τ_{NH} = ^{15}N-^{1}H cross polarization contact time; $\tau_{MISSISSIPPI}$ = duration of MISSISSIPPI solvent suppression period, τ_{REDOR} = ^{15}N-^{13}C REDOR mixing time; ν_C = ^{13}C rf field strength for 180° pulses during REDOR.

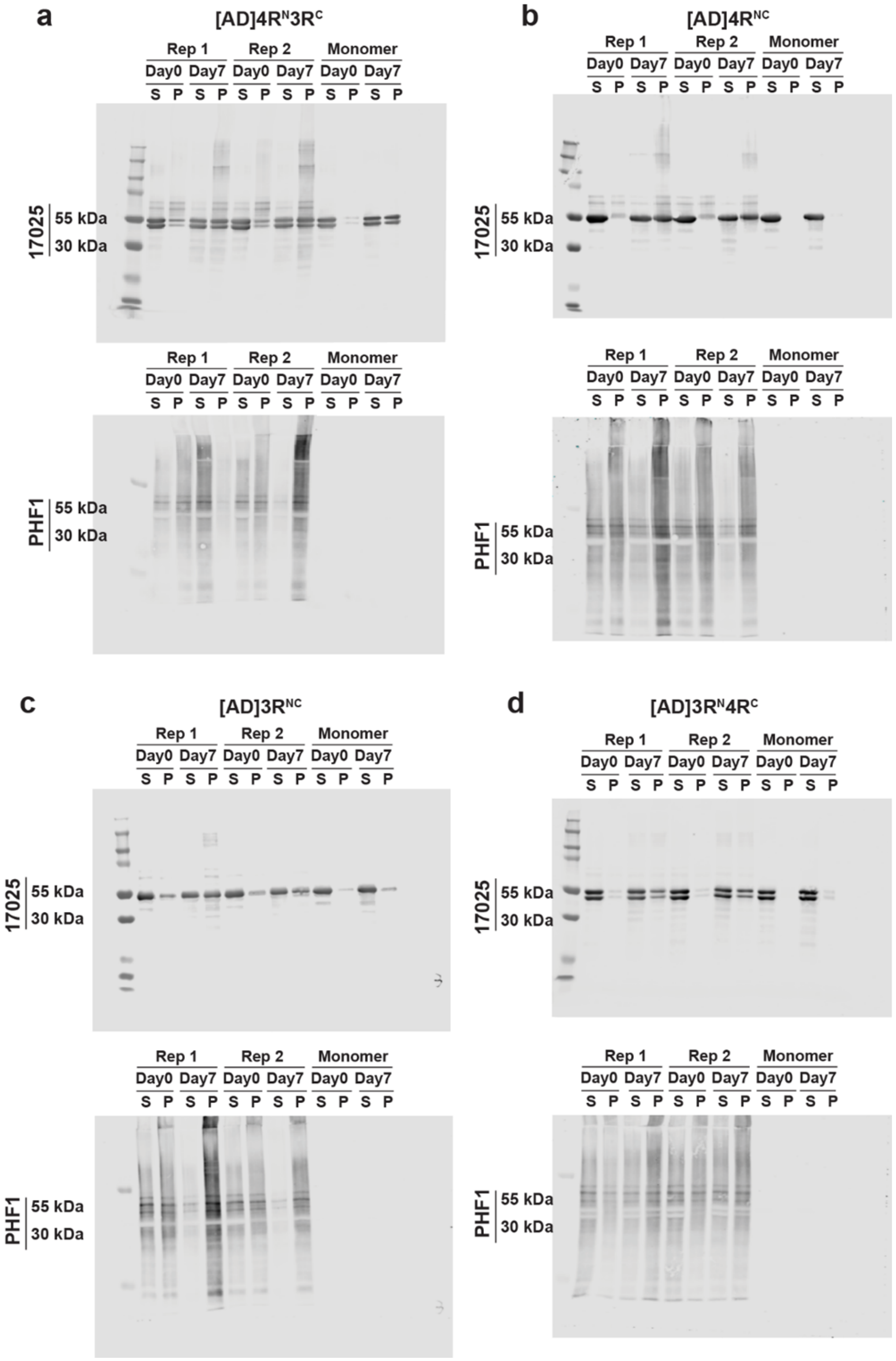

Supplementary Fig. 1. Full Western Blot images from Fig. 2a. Tau species in (**a**) [AD]4R^{N}3R^{C}, (**b**) [AD]4R^{NC}, (**c**) [AD]3R^{NC} and (**d**) [AD]3R^{N}4R^{C} were revealed by the 17025 anti-total tau antibody and PHF1 anti-phospho-tau antibody.

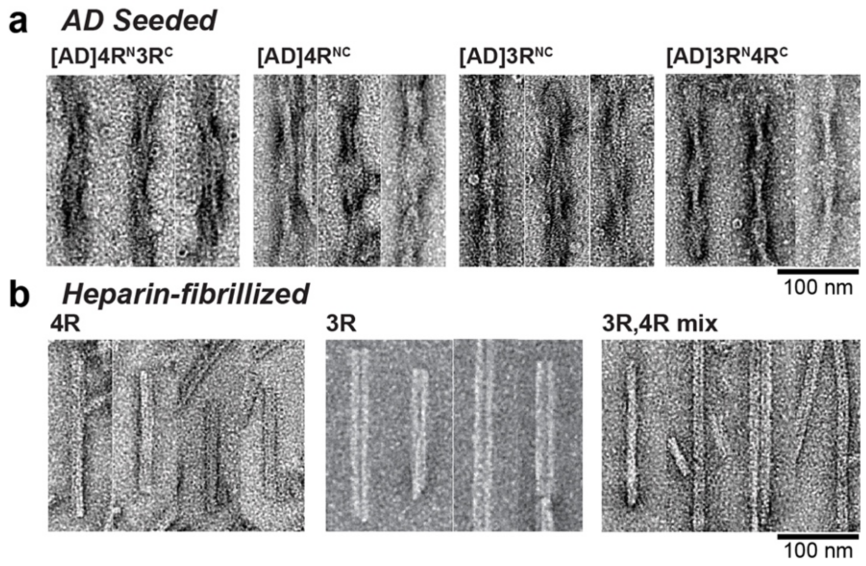

Supplementary Fig. 2. **TEM Images of tau filaments aligned for comparison.** (**a**) The majority of fibrils in AD-seeded samples are twisted filaments that have uniform width and crossover distance, and that match the dimensions previously reported for AD paired-helical filaments (PHF) [45]. (**b**) Heparin-fibrillized tau containing 4R tau only, 3R tau only, and both 4R and 3R tau. These fibrils are nearly exclusively straight and are distinct from AD seeded tau filaments. At least five different images from different regions of each grid were obtained for each sample.

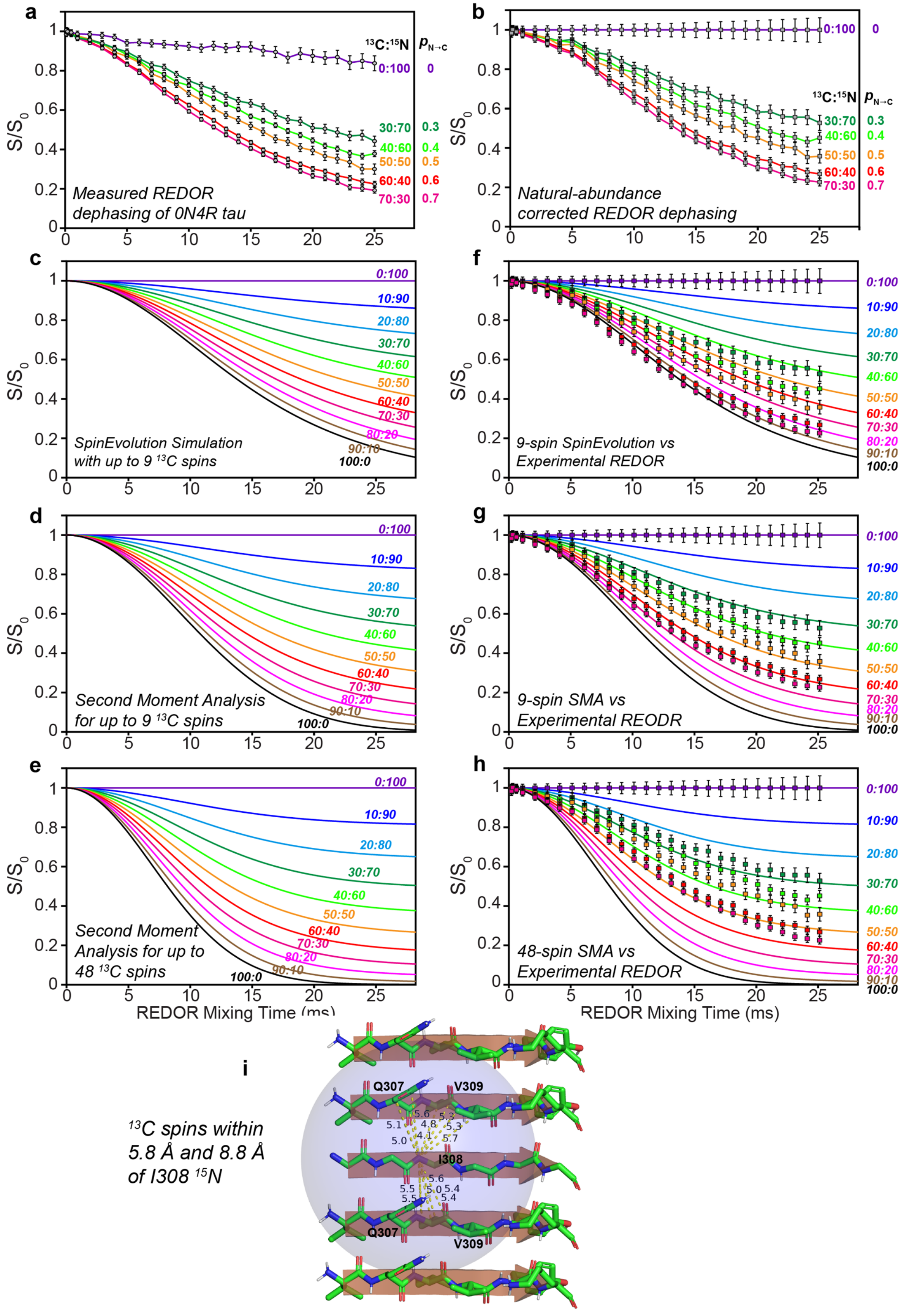
a
b
c
d
e
f
g
h
i
S/S0
REDOR Mixing Time (ms)
13C:15N
pN→C
0:100
10:90
20:80
30:70
40:60
50:50
60:40
70:30
80:20
90:10
100:0
0
0.3
0.4
0.5
0.6
0.7
Measured REDOR dephasing of 0N4R tau
Natural-abundance corrected REDOR dephasing
SpinEvolution Simulation with up to 9 13C spins
9-spin SpinEvolution vs Experimental REDOR
Second Moment Analysis for up to 9 13C spins
9-spin SMA vs Experimental REODR
Second Moment Analysis for up to 48 13C spins
48-spin SMA vs Experimental REDOR
13C spins within 5.8 Å and 8.8 Å of I308 15N
Q307
V309
I308

Supplementary Fig. 3. Measured and simulated ^{15}N-^{13}C REDOR dephasing curves of mixed labeled 0N4R tau with varying ratios of ^{13}C, ^{2}H-labeled protein and ^{15}N, ^{2}H-labeled protein. (**a**) Measured REDOR dephasing of ^{13}C and ^{15}N mixed labeled 0N4R tau with varying mixing ratios, reproduced from Fig. 3e. Natural abundance ^{13}C dephasing to ^{15}N-labeled protein is present in these data. Error bars represent the propogated uncertainty from the spectral signal-to-noise ratios. (**b**) ^{13}C natural-abundance corrected REDOR data, obtained by dividing the measured S/S_0 values of each sample by the S/S_0 values of the 100% ^{15}N-labeled tau. Error bars represent the propogated uncertainty from the spectral signal-to-noise ratios. (**c**) SpinEvolution simulation using up to 9 nearest ^{13}C spins to the I308 amide ^{15}N. The natural abundance ^{13}C nuclei in the ^{15}N-labeled monomer are ignored in these simulations. (**d**) Second moment analysis (SMA) of REDOR dephasing using the same ^{13}C spins as in (**b**). (**e**) Second moment analysis of REDOR dephasing using the nearest 48 ^{13}C spins within 8.8 Å of the ^{15}N nucleus. The second moment simulations ignore ^{13}C finite-pulse effects, ^{13}C isotropic and anisotropic chemical shifts, and ^{13}C-^{13}C dipolar couplings. (**f-h**) overlay of the measured natural-abundance corrected REDOR data in (**b**) with the SpinEvolution simulation (**c**) and second moment analysis (**d-e**). (**i**) ^{13}C spins within 5.8 Å (dashed lines) or 8.8 Å (purple sphere) of I308 amide ^{15}N in the cryoEM structure of AD PHF tau (PDB: 5O3L) [4]. The two neighboring chains that sandwich the central chain with an I308 ^{15}N label are ^{13}C-labeled either in one or both chains.

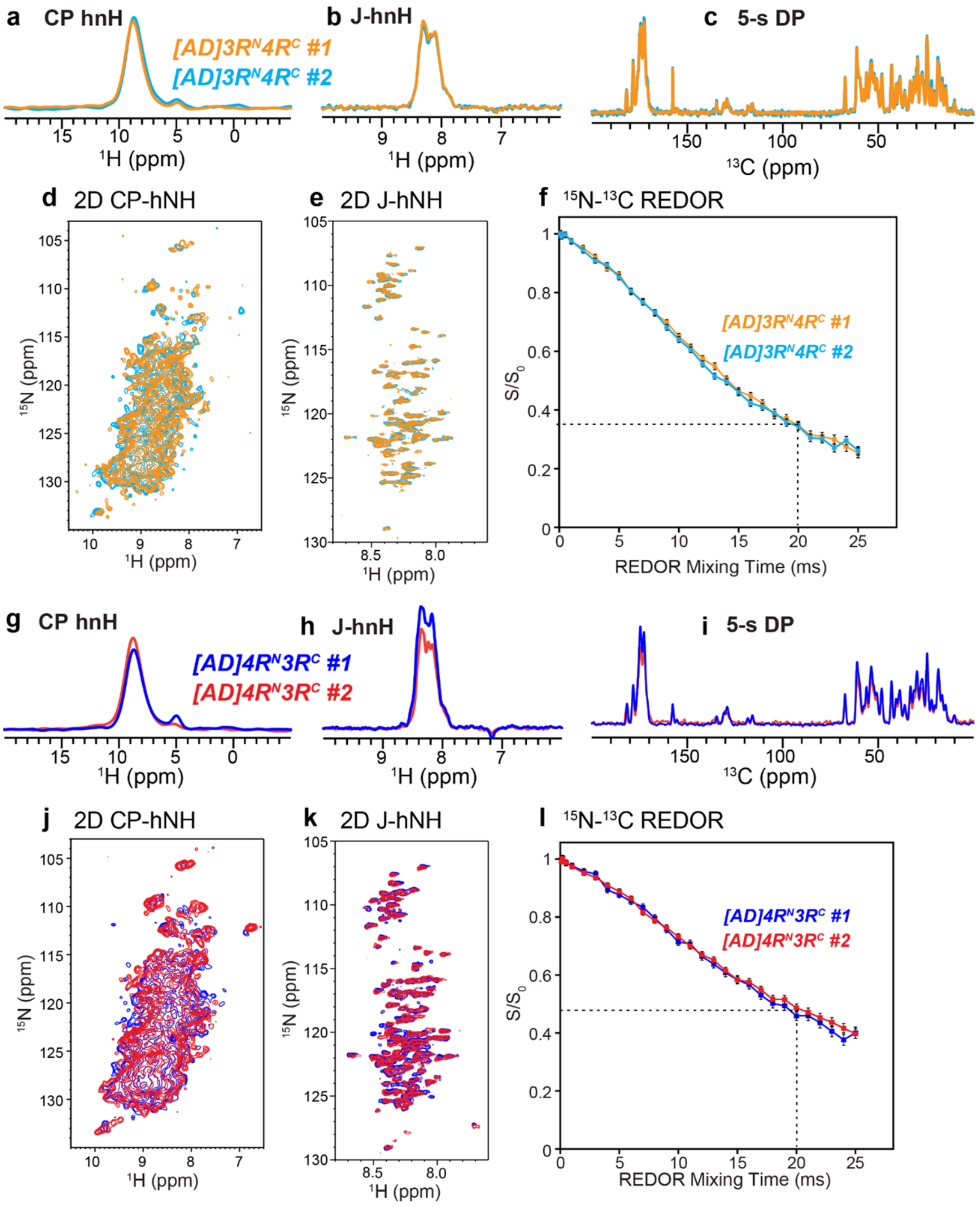

Supplementary Fig. 4. AD seeded mixed isoform tau filaments have highly reproducible structure and dynamics. (**a-f**) Comparisons of the fingerprint spectra and REDOR data of two independent samples of AD-seeded ^{15}N-labeled 3R tau mixed with ^{13}C-labeled 4R tau (1:1). (**g-l**) Comparisons of fingerprint spectra and REDOR data of two independent AD seeded ^{15}N-labeled 4R tau mixed with ^{13}C-labeled 3R tau (1:1). Each sample was seeded separately using separately

purified tau monomers. (**a-c**) 1D CP-hnH, J-hnH, and ^{13}C DP MAS spectra of duplicates #1 and #2 of [AD]3R^N4R^C tau. (**d-e**) 2D CP-hNH and J-hNH spectra of the same duplicates. (**f**) ^{1}H-detected ^{15}N-^{13}C REDOR dephasing of the duplicates. Similar comparisons between the duplicates of [AD]4R^N3R^C are shown in (**g-l**).

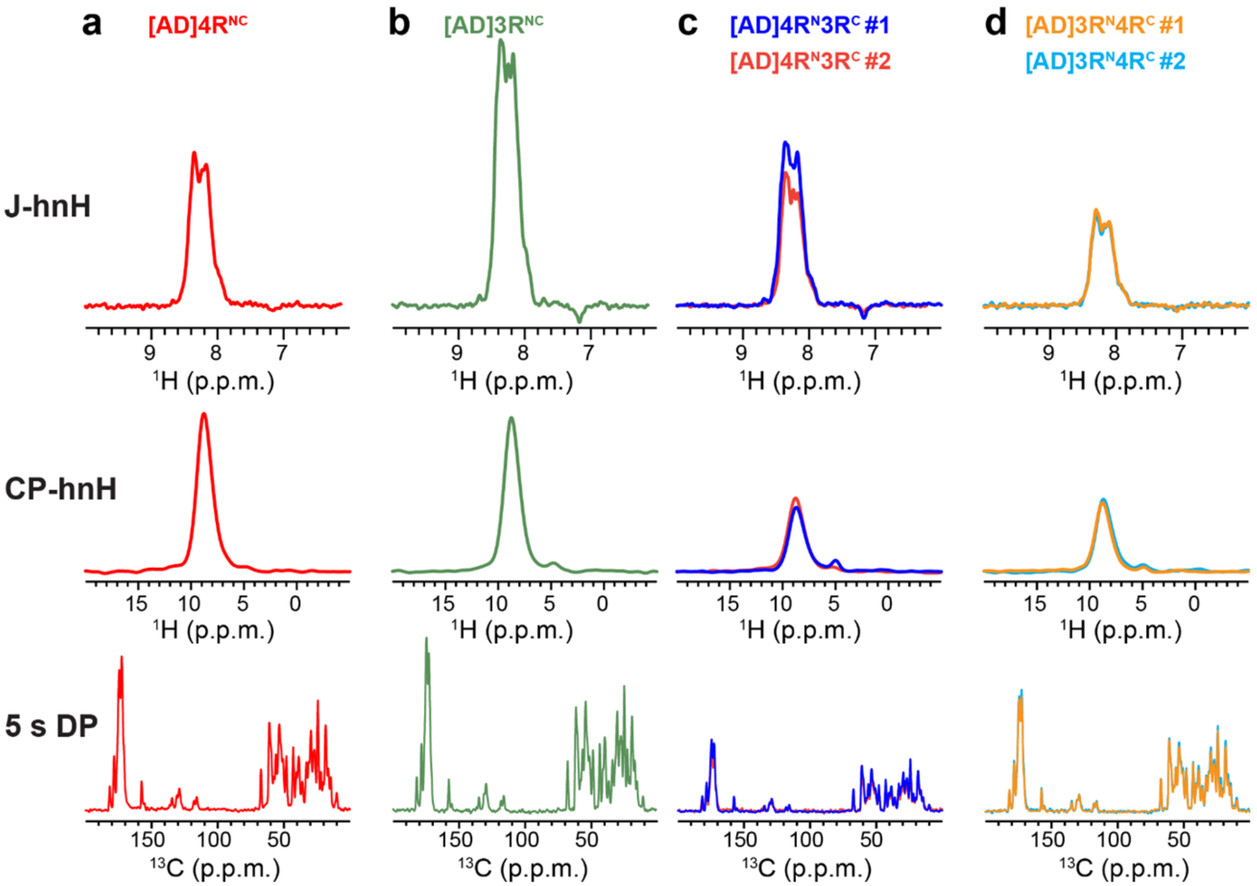

Supplementary Fig. 5. 1D ^{1}H-detected J-hnH spectra, ^{1}H-detected CP-hnH spectra, and ^{13}C DP-MAS spectra of AD-tau seeded tau filaments for quantification of 4R and 3R tau incorporation levels into the filaments. (**a**) [AD]4R^{NC} sample. (**b**) [AD]3R^{NC} sample. (**c**) Duplicates #1 and #2 of [AD]4R^{N}3R^C sample, which contains a 1:1 mixture of ^{15}N, ^{2}H-labeled 0N4R tau and ^{13}C, ^{2}H-labeled 0N3R tau. (**d**) Duplicates #1 and #2 of [AD]4R^{N}3R^C sample, which contains a 1:1 mixture of ^{15}N, ^{2}H-labeled 0N3R tau and ^{13}C, ^{2}H-labeled 0N4R tau.

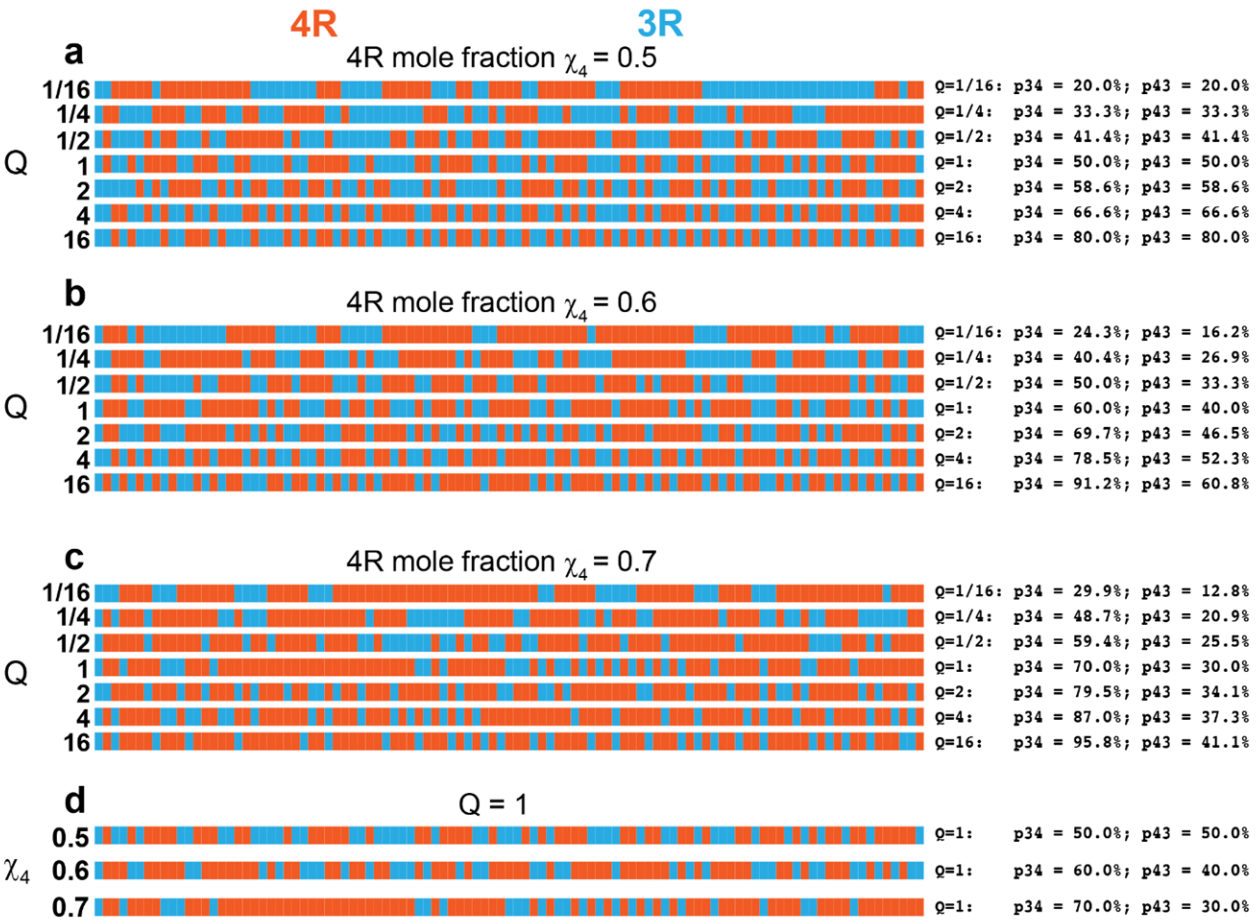

Supplementary Fig. 6. Simulated fibril mixing schemes for varying mole fractions of 4R tau (χ_4) and mixing quotients Q. Individual 100-monomer fibrils made of 3R (blue) or 4R (orange) monomers are shown; each monomer is a vertical line. (**a**) Simulated mixing as a function of Q for $\chi_4 = 0.5$. (**b**) Simulated mixing as a function of Q for $\chi_4 = 0.6$. (**c**) Simulated mixing as a function of Q for $\chi_4 = 0.7$. (**d**) Simulated mixing as a function of χ_4 for Q = 1.

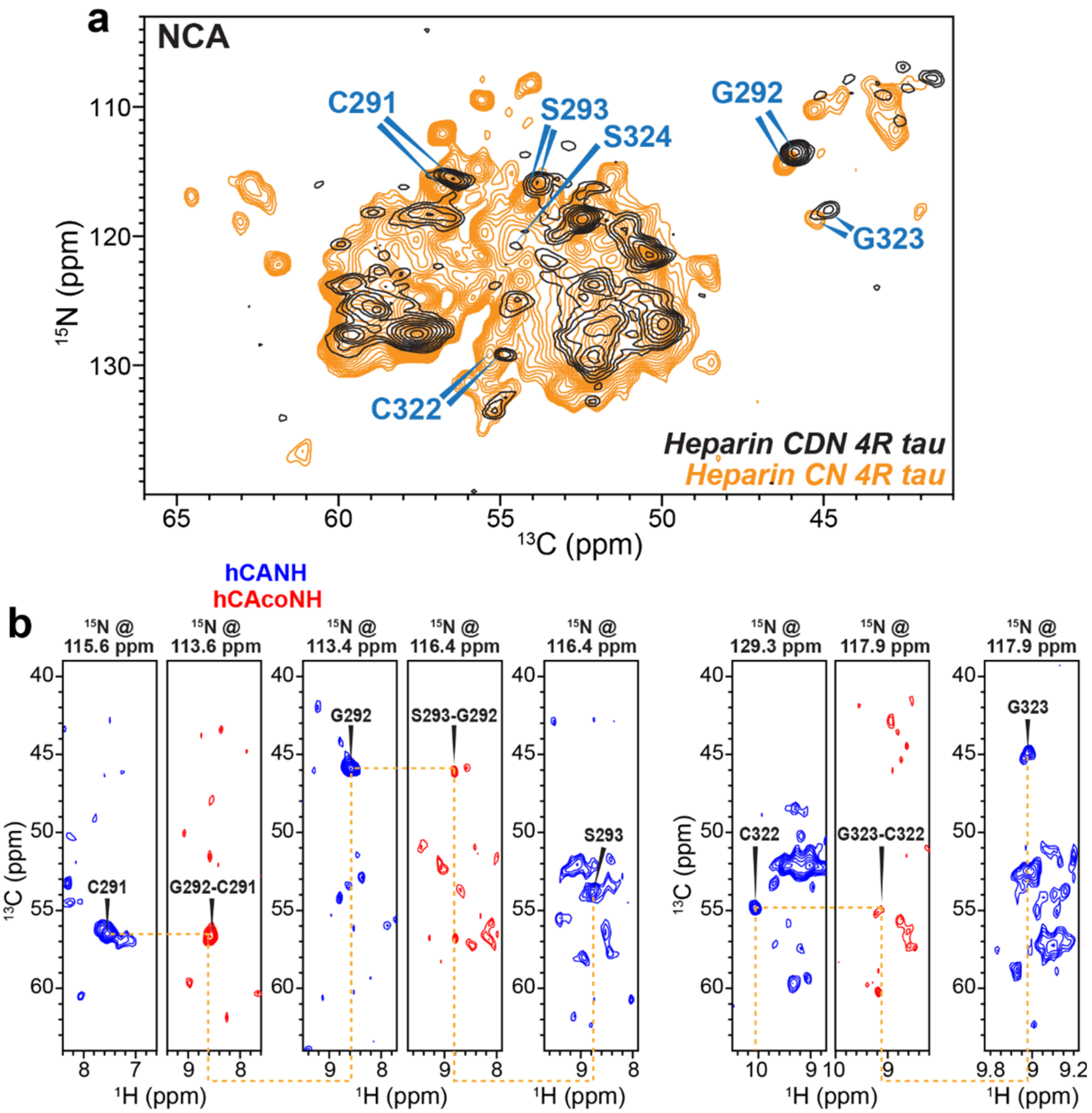

Supplementary Fig. 7. Assignment of residues ^{291}CGS^{293} and ^{322}CGS^{294} in heparin-fibrilized 4R^{NC} tau based on 3D hCANH and hCA(co)NH spectra. (**a**) 2D NC projection from the 3D hCANH spectrum of the heparin-fibrillized CDN-labeled 4R tau (black), overlaid with the previously reported 2D NCA spectrum of heparin-fibrillized CN-labeled 4R tau (orange) [21]. Residues ^{291}CGS^{293} and ^{322}CGS^{294} in both spectra are marked in cyan. The small chemical shift differences between the two samples can be attributed to deuterium isotope effects and slight difference in experimental temperatures. (**b**) CAH strips extracted at the ^{15}N chemical shifts of residues ^{291}CGS^{293} and ^{322}CG^{293} from 3D the hCANH (blue) and hCA(co)NH (red) spectra of the CDN-labeled 4R tau sample. Residues ^{291}CGS^{293} and ^{322}CG^{323} have the strongest peak intensities in these 3D ^{1}H-detected spectra, consistent with the observation of CN-labeled 4R tau sample in previously reported ^{13}C-detected 3D spectra [21].
