# Supplementary material for: Fluent molecular mixing of Tau isoforms in Alzheimer’s disease neurofibrillary tangles
Source: Nat Commun. 2022 May 27;13:2967. doi: 10.1038/s41467-022-30585-0 (PMC9142584; doi:10.1038/s41467-022-30585-0)
Supplement: Supplementary file 3 — Supplementary Software [file 41467_2022_30585_MOESM3_ESM.pdf]

Source Codes for

Fluent Molecular Mixing of Tau Isoforms in Alzheimer's Disease Neurofibrillary Tangles

Aurelio J. Dregni [1$], Pu Duan[1$], Hong Xu[2], Lakshmi Changolkar[2], Nadia El Mammeri[1], Virginia M.-Y. Lee[2], and Mei Hong[1]*

[1] Department of Chemistry, Massachusetts Institute of Technology, 170 Albany Street, Cambridge, MA 02139

[2] Department of Pathology and Laboratory Medicine, Institute On Aging and Center for Neurodegenerative Disease Research, University of Pennsylvania School of Medicine, Philadelphia, PA, 19104, USA

* Corresponding author: Mei Hong: meihong@mit.edu
[$] These authors contributed equally to this work.

SpinEvolution Source Code for REDOR simulation

SpinEvolution Code for the case of ^{13}C-chain → ^{15}N-chain → ^{13}C-chain:

```
****** The System ***********************************
spectrometer(MHz)  600
spinning_freq(kHz) 20.0
channels          N15 C13
nuclei            N15 C13 C13 C13 C13 C13 C13 C13 C13 C13
atomic_coords     I308_5p55_343.txt
cs_isotropic      0 -47 70 -65 -40 70 -82 -40 -62 -75 ppm
csa_parameters    1 0 0 0 0 0 ppm
csa_parameters    2 30 0 10 45 0 ppm
csa_parameters    3 170 0 90 30 0 ppm
csa_parameters    4 30 0 50 10 0 ppm
csa_parameters    5 30 0 80 60 0 ppm
csa_parameters    6 170 0 5 20 0 ppm
csa_parameters    7 30 0 49 10 0 ppm
csa_parameters    8 30 0 25 60 0 ppm
csa_parameters    9 30 0 50 20 0 ppm
csa_parameters    10 30 0 30 20 0 ppm
j_coupling        *
quadrupole        *
dip_switchboard   *
csa_switchboard   *
exchange_nuclei   *
bond_len_nuclei   *
bond_ang_nuclei   *
tors_ang_nuclei   *
groups_nuclei     *
******* REDOR Pulse Sequence **********************
CHN 1
timing(usec)     (50)512    42    8    50      (50)512
power(kHz)         0         0   62.5   0        0
phase(deg)         0         0    0     0        0
freq_offs(kHz)     0         0    0     0        0
CHN 2
timing(usec)    (redor.pp)   17  8    25  redor.pp (redor.pp)
power(kHz)         *          0  62.5  0     *         *
phase(deg)         *          0  0     0     *         *
freq_offs(kHz)     *          0  0     0     *         *

******* Variables ***********************************
scan_par1d rf/62.5:-1.74:52.06/
power_2_1_2 = rf
power_2_1_4 = rf
power_2_2_2 = rf
power_2_2_5 = rf
power_2_2_7 = rf
power_2_3_2 = rf
power_2_3_4 = rf
******* Options *************************************
rho0              I1x
observables       I1p
EulerAngles       rep30
```

n_gamma	*
line_broaden(Hz)	*
zerofill	*
FFT_dimensions	*
options	-dw123 -re

“atomic_coords” file for the case of ^{13}C-chain → ^{15}N-chain → ^{13}C-chain:

130.013103.502142.851	N	ILE	F	0
129.061101.533138.265	CA	GLN	below	5.080813025
130.095102.462138.877	C	GLN	below	4.108649413
127.663102.083138.528	CB	GLN	below	5.120975493
131.652104.3 138.441	CA	ILE	below	4.771920473
130.98 105.609138.057	C	ILE	below	5.325126665
128.329107.077139.319	CG2	VAL	below	5.300141979
130.205104.843148.098	CA	ILE	Above	5.419054715
131.602104.671147.465	CB	ILE	above	5.018015345
132.221103.35 147.919	CG1	ILE	above	5.530189147

SpinEvolution Code for the case of ^{13}C-chain → ^{15}N-chain → ^{15}N-chain:

```
****** The System ***********************************
spectrometer(MHz)  600
spinning_freq(kHz) 20.0
channels          N15 C13
nuclei            N15 C13 C13 C13 C13 C13
atomic_coords     I308_5p8_344.txt
cs_isotropic      0 -40 70 -60 -85 -90 ppm
csa_parameters    1 0 0 0 0 0 ppm
csa_parameters    2 30 0 10 45 0 ppm
csa_parameters    3 170 0 90 30 0 ppm
csa_parameters    4 30 0 50 10 0 ppm
csa_parameters    5 30 0 80 60 0 ppm
csa_parameters    6 30 0 5 20 0 ppm
j_coupling        *
quadrupole        *
dip_switchboard   *
csa_switchboard   *
exchange_nuclei   *
bond_len_nuclei   *
bond_ang_nuclei   *
tors_ang_nuclei   *
groups_nuclei     *
******* REDOR Pulse Sequence **********************
CHN 1
timing(usec)      (50)512     42     8    50       (50)512
power(kHz)        0           0    62.5   0         0
phase(deg)        0           0     0     0         0
freq_offs(kHz)    0           0     0     0         0
CHN 2
timing(usec)    (redor.pp)   17  8     25  redor.pp (redor.pp)
power(kHz)        *           0   62.5  0     *           *
phase(deg)        *           0   0     0     *           *
freq_offs(kHz)    *           0   0     0     *           *
******* Variables ***********************************
scan_par1d rf/62.5:-1.74:52.06/
power_2_1_2 = rf
power_2_1_4 = rf
power_2_2_2 = rf
power_2_2_5 = rf
power_2_2_7 = rf
power_2_3_2 = rf
power_2_3_4 = rf
******* Options *************************************
rho0              I1x
observables       I1p
EulerAngles       rep30
n_gamma           *
line_broaden(Hz)  *
zerofill          *
FFT_dimensions    *
options           -dw123 -re
```

“atomic_coords” file for the case of ^{13}C-chain → ^{15}N-chain → ^{15}N-chain:

130.013103.502142.851		N	ILE	F	0	
130.205104.843148.098		CA	ILE	Above	5.419054715	
129.585106.179147.718		C	ILE	above	5.571104199	
131.602104.671147.465		CB	ILE	above	5.018015345	
132.221103.35	147.919		CG1	ILE	above	5.530189147
133.458102.93	147.167		CD1	ILE	above	5.551852394

SpinEvolution Code for the case of ^{15}N-chain → ^{15}N-chain → ^{13}C-chain:

```
****** The System **********************************
spectrometer(MHz)  600
spinning_freq(kHz) 20.0
channels          N15 C13
nuclei            N15 C13 C13 C13 C13 C13 C13 C13 C13
atomic_coords      I308_5p8_443.txt
cs_isotropic      0 -47 70 -65 -67 -40 75 -70 -82 ppm
csa_parameters    1 0 0 0 0 0 ppm
csa_parameters    2 30 0 10 45 0 ppm
csa_parameters    3 170 0 90 30 0 ppm
csa_parameters    4 30 0 50 10 0 ppm
csa_parameters    5 30 0 80 60 0 ppm
csa_parameters    6 30 0 5 20 0 ppm
csa_parameters    7 170 0 49 10 0 ppm
csa_parameters    8 30 0 25 60 0 ppm
csa_parameters    9 30 0 50 20 0 ppm
j_coupling        *
quadrupole        *
dip_switchboard    *
csa_switchboard    *
exchange_nuclei    *
bond_len_nuclei    *
bond_ang_nuclei    *
tors_ang_nuclei    *
groups_nuclei      *
******* REDOR Pulse Sequence **********************
CHN 1
timing(usec)      (50)512     42     8    50       (50)512
power(kHz)         0          0    62.5   0         0
phase(deg)        0           0     0     0         0
freq_offs(kHz)     0          0     0     0         0
CHN 2
timing(usec)    (redor.pp)   17  8     25  redor.pp (redor.pp)
power(kHz)         *           0   62.5  0     *           *
phase(deg)         *           0  0      0     *           *
freq_offs(kHz)     *          0  0       0     *           *
******* Variables ***********************************
scan_par1d rf/62.5:-1.74:52.06/
power_2_1_2 = rf
power_2_1_4 = rf
power_2_2_2 = rf
power_2_2_5 = rf
power_2_2_7 = rf
power_2_3_2 = rf
power_2_3_4 = rf
******* Options ************************************
rho0             I1x
observables       I1p
EulerAngles       rep30
n_gamma           *
line_broaden(Hz)   *
zerofill          *
FFT_dimensions     *
options          -dw123 -re
```

“atomic_coords” file for the case of ^{13}C-chain → ^{15}N-chain → ^{15}N-chain:

130.013103.502142.851	N	ILE	F	0
129.061101.533138.265	CA	GLN	below	5.080813025
130.095102.462138.877	C	GLN	below	4.108649413
127.663102.083138.528	CB	GLN	below	5.120975493
127.402103.439137.901	CG	GLN	below	5.59676603
131.652104.3 138.441	CA	ILE	below	4.771920473
130.98 105.609138.057	C	ILE	below	5.325126665
129.384108.127139.63	CB	VAL	below	5.671076353
128.329107.077139.319	CG2	VAL	below	5.300141979

SpinEvolution pulse sequence file "redor.pp":

```
17   0      0   0
8    62.5   0   0
17   0      0   0
8    62.5   90  0
```

Bruker Topspin 3.5 pulse program code for ^{1}H-detected ^{15}N-^{13}C REDOR

```
;1H detected 15N{13C} REDOR
;Avance III version
;f1 : H
;f2 : N
;f3 : C
;o1 : H offset, on resonance with water peak (~5 ppm, water suppression!)
;o2 : N offset, center of 15N signal (~119 ppm)
;o3 : C offset, center of 13C signal (~100 ppm, using '-DTC')
;p3 : H 90 hard pulse at pl2
;p21 : N 90 hard pulse at pl21
;p22 : N 180 hard pulse at pl21
;p33 : C 180 hard pulse at pl3 for redor
;p25 : HN CP at sp42 (H) & sp43 (N), (~1 to 3 ms)
;p45 : NH CP at sp46 (H) & sp47 (N), (~400 to 800 us)
;pl2 : H hard pulse power
;pl3 : C hard pL for REDOR
;pl12 : H dec power ('tppm15' at >70 kHz for <14 kHz MAS; 'waltz16_12nofq' at ~10 kHz for fast MAS)
;pl13 : H dec power during H2O suppression (~15 kHz, 'cwX_13nofq', 'cwY_13nofq')
;pl16 : N dec power ('waltz16_16nofq' at ~7 kHz)
;pl18 : C dec power ('waltz16_18nofq' at ~7 kHz, using '-DTC')
;pl21 : N hard pulse power (can be optimized with '-DN90')
;sp42 : H HN CP power
;sp43 : N HN CP power
;sp46 : H NH CP power
;sp47 : N NH CP power
;d1 : recycle delay; 1 to 5 times T1 (~0.8 to 1 s)
;d19 : delay for water suppression (~100 to 300 ms)
;d5: Total REDOR time, tune by l10
;cpdprg1 : H dec ('tppm15' at >70 kHz for <14 kHz MAS; 'waltz16_12nofq' at ~10 kHz for fast MAS)
;cpdprg2 : N dec ('waltz16_16nofq' at pl16 (10 kHz))
;cpdprg3 : C dec ('waltz16_18nofq' at pl18 (10 kHz), using '-DTC')
;cpdprg4 : H Water suppression along X ('cwX_13nofq' at pl13 (15 kHz))
;cpdprg5 : H Water suppression along Y ('cwY_13nofq' at pl13 (15 kHz))
;pcpd1 : H dec pulse
;pcpd2 : N dec pulse: 35.35 us ('waltz16_16nofq' ~7 kHz)
;pcpd3 : C dec pulse: 35.35 us ('waltz16_18nofq' ~7 kHz, using '-DTC')
;spnam42 : H shape (ramp up for NH CP, e.g. 'ramp.70100.1000')
;spnam43 : N shape (e.g. 'square.1000' for HN CP (=no shape))
;spnam46 : H shape (ramp down for NH CP, e.g. 'ramp.10070.1000')
;spnam47 : N shape (e.g. 'square.1000' for NH CP (=no shape))
;inf1 :  1/SW(N) = 2 * DW(N)
;in0 : = inf1
;l31: spinning speed in Hz
;l10 : REDOR mix = L10*tr, must be even
;ZGOPTNS : -DTC : switch on C decoupling
;  -DN90: N 90 degree pulse check
;  or blank
;FnMODE : States-TPPI
;ns : MIN. 4 (full: 8)
;ds : 2 or 4

;##########################################################
;# hNHREDOR                                   #
;# Dregni, Duan… Hong, Nature Communications 2022                #
```

```
;#######################################################

;$COMMENT=1H detected 15N{13C} REDOR
;$CLASS=BioSolids
;$DIM=1D
;$TYPE=H detection
;$SUBTYPE=CP, Heteronuclear

prosol relations=<biosolHCN>

#include <HNC_defs.incl>
	; defines H:f1, N:f2, C:f3

#include <trigg.incl>
	; definition of external trigger output

"acqt0=-(p1*2/3.1416)-0.5u"     ; baseopt correction

"spoff42=0.0"           ;###########################
"spoff43=0.0"           ;#     ensure correct     #
"spoff46=0.0"           ;#     shape offsets      #
"spoff47=0.0"           ;###########################

"p2=p1*2"
"p22=p21*2"

  "d11=0.5s/l31-p22/2"                  ;to center square refocusing pulse p22
  "d25=0.25s/l31-1u"                    ;REDOR. Used at beginning and end.
  "d26=d25-p21/2"                       ;Account for N flip up
  "d27=0.25s/l31-p33/2"                 ;REDOR, altered for square timing
  "d28=0.25s/l31"                       ;Tr/4, REDOR
  "d5=l10/l31"

"in0=inf1"              ;###########################
"d0=1u"                 ;#    t1_init => 0, 0     #
"l0=0"                  ;###########################

                  ;###########################
;$EXTERN                ;# python insertion point #
                  ;###########################

Prepare, ze

;#######################################################
;#          Start of Active Pulse Program            #
;#######################################################

Start, 30m  do:N

#ifdef TC
  0.5u do:C
#endif          /* end of TC */

  d1
  d5
```

```
  trigg

if "l0>0"
{
  "d51=d0-1u"
}

;######################################################
;#            Initial excitation & HN CP            #
;######################################################

  (p3 pl2 ph1):H

  (p25:sp42 ph0):H  (p25:sp43 ph2):N

;------------NC REDOR------------------------------

  1u cpds1:H                                ;composite pulse decoupling scheme for REDOR

  d25 pl3:C

6 d27
  p33:C ph15^
  d27
lo to 6 times l10                    ; L0 still must be even


  d28
  d11
  (p22 pl21 ph10):N                  ;square pulse - for nonselective appplications
  d11
  d28

8 d27
  p33:C ph15^
  d27
lo to 8 times l10

  d26
  1u do:H

;----------End of REDOR------------------------------

#ifdef TC
if "l0>0"
{
  1u cpds1:H
  (center (d51) (p33 pl3 ph20):C)
  0.5u do:H  pl13:H
}
#else
if "l0>0"
{
  0.5u cpds1:H
  d51
```

```
  0.5u do:H  pl13:H
}
#endif         /* end of TC */


;#######################################################
;#            Water suppression                     #
;#######################################################

  (p21 pl21 ph3):N          ; brings magn. to z

  0.5u cpds4:H
  d19*0.25
  0.5u do:H

  0.5u cpds5:H
  d19*0.25
  0.5u do:H

  0.5u cpds4:H
  d19*0.25
  0.5u do:H

  0.5u cpds5:H
  d19*0.25
  0.5u do:H

  (p21 pl21 ph4):N          ; brings magn. to y

;#######################################################
;#          15N hard pulse optimization             #
;#######################################################

#ifdef N90                  ; brings magn. to z.
  (p21 pl21 ph22):N
#endif          /* end of N90 */

;#######################################################
;#                    N-H CP                         #
;#######################################################

  (p45:sp47 ph5):N  (p45:sp46 ph6):H

;########################################################
;#                    Acquisition                    #
;########################################################

#ifdef TC
  1u cpds2:N  cpds3:C
#else
  0.5u pl16:N
  0.5u cpds2:N
#endif         /* end of TC */

  gosc ph31     ;start ADC with ph31 signal routing
```

```
#ifdef TC
  1m do:C  do:N
#else
  1m do:N
#endif         /* end of TC */

lo to Start times ns

30m mc #0 to Start
F1PH(calph(ph2, +90), caldel(d0, +in0) & calclc(l0, 1))

HaltAcqu, 1m
exit

;######################################
;#            Phase Cycle           #
;######################################

ph1 = 1 3                      ; 1H 90 hard pulse
ph0 = 0                        ; 1H HN CP Spin lock
ph2 = 1 1 1 1 3 3 3 3          ; 15N HN CP Spin lock
ph3 = 0                        ; 15N 1st 90 hard pulse (flip to z)
ph4 = 0 0 2 2                  ; 15N 2nd 90 hard pulse (flip back)
ph5 = 1                        ; 15N NH CP Spin lock
ph6 = 1                        ; 1H NH CP Spin lock
ph10= 1 1 1 1 1 1 1 1
      3 3 3 3 3 3 3 3          ;F1 180, normal shaped pulse

ph15=  0 1 0 1                 ; xy-4. F3 phase
ph31= 1 3 3 1 3 1 1 3          ; receiver

#ifdef TC
ph20= 0                 ; C 180 hard pulse
#endif                  /* end of TC */

#ifdef N90
ph22= 0 0 2 2           ;N hard pulse
#endif                  /* end of N90 */

;######################################################
```

Python Source Code for Building Fibril Model (Fig 3f, g)

```
#Simulates a N residue fibril with given p34 and p43.
#Then computes observed probabilities in the simulated fibril.
#Returns "results", a string of '3', and '4' indicating the simulated fibril.
#Also returns obsProbs: (p43 observed in sim fibril - input p43, p34 observed in sim fibril - p34)
#Repeat simulation until observed probabilities are within one percentage point of input probabilities

from random import random

p34 = 0.56
p33 = 1-p34
p43 = 0.37
p44 = 1-p43

results = '3'
N = 360
for idx in range(N):
    r = random()
    if results[-1] == '3':
        if r < p34:
            results = results + '4'
        else:
            results = results + '3'
    else:
        if r < p43:
            results = results + '3'
        else:
            results = results + '4'

print(results)
obs4 = results.count('4')
obs3 = results.count('3')
obs43 = results.count('43')
obs44 = obs4 - obs43
obs34 = results.count('34')
obs33 = obs3 - obs34
#obsProbs = (obs4/N,obs44/obs4,obs43/obs4,obs34/obs3,obs33/obs3) #p4, p44, p43, p34, p33
obsProbs = (obs43/obs4-p43,obs34/obs3-p34)
obsProbs
```

Python Source Code for Fibril Models with Varying Q and χ_4 (Supplementary Fig. 7)

```
#Simulates an N residue fibril with given chi4 and Q.
#First computes p34 and p43 from chi4 and Q (A quadratic equation)
#Then simulates fibril based on p34 and p43.
#Variable "Results" is printed and contains the simulated fibril.
#Also returns obsParams: (Chi4 observed in sim fibril, input Chi4, Q observed in sim fibril, input Q)
#Repeat simulation until observed and input parameters are within appropriate tolerance.

from random import random
import math as mat
chi4 = 0.7 #macroscopic preference = p34 / (p43 + p34)
Q = 1/16 #microscopic mixing parameter = p34 p43 / (p33 p44).
        #Choose slightly off 1.0, as equations fail for Q=1 exactly
W = (1-chi4)/chi4
p34 = 1/(2*W*(Q-1)) * (Q*(1+W) - math.sqrt((Q**2) *((1+W)**2)-4*Q*W*(Q-1)))
p43 = W*p34

p33 = 1-p34
p44 = 1-p43
probs = (p34, p43, p33, p44)
probs
results = '3'
N = 100
for idx in range(N):
  r = random()
  if results[-1] == '3':
    if r < p34:
      results = results + '4'
    else:
      results = results + '3'
  else:
    if r < p43:
      results = results + '3'
    else:
      results = results + '4'

print(results)
obs4 = results.count('4')
obs3 = results.count('3')
obs43 = results.count('43')
obs44 = obs4 - obs43
obs34 = results.count('34')
obs33 = obs3 - obs34
obsParams = (obs4/N, chi4, obs43*obs34/(obs44*obs33),Q)
obsParams
```
